# Circ_0084043 promotes cell proliferation and glycolysis but blocks cell apoptosis in melanoma via circ_0084043-miR-31-KLF3 axis

**DOI:** 10.1515/biol-2020-0071

**Published:** 2020-10-22

**Authors:** Songjiang Wu, Yuhan Tang, Wenli Liu

**Affiliations:** Department of Dermatology, The First Affiliated Hospital of University of South China, Hengyang 421001, Hunan, China; Department of Oncology, Chinese Medicine Hospital of Hengyang, Hengyang 421000, Hunan, China; Department of Oncology, Affiliated Nanhua Hospital, University of South China, No. 336, Dongfang South Road, Zhuhui District, Hengyang 421000, Hunan, China

**Keywords:** circ_0084043, miR-31, KLF3, melanoma

## Abstract

Melanoma is an aggressive malignant tumor. The crucial role of circular RNAs has been documented in many types of cancer, including melanoma. The objective of this study was to uncover the function of circ_0084043 in the biological process of melanoma and associated mechanism of action. The expression of circ_0084043, miR-31, and Krüppel-like factor 3 (KLF3) was determined by qRT-PCR. Cell proliferation and apoptosis were monitored by the MTT assay and flow cytometry assay, respectively. The progression of glycolysis was evaluated according to the levels of glucose consumption, lactate production, and ATP concentration using appropriate detection kits. The relationship between miR-31 and circ_0084043 or KLF3 was predicted by the bioinformatics tool and ascertained by the dual-luciferase reporter assay. The protein levels of KLF3 and glucose transporter 1 (Glut1) were quantified by western blot. A xenograft model was established to ascertain the role of circ_0084043 *in vivo*. As a result, circ_0084043 expression was reinforced in melanoma tissues and cells. Circ_0084043 knockdown inhibited cell proliferation, induced cell apoptosis, and restrained glycolysis. MiR-31 was a target of circ_0084043, and miR-31 deficiency reversed the role of circ_0084043 knockdown. KLF3 was targeted by miR-31, and KLF3 upregulation abolished the effects of miR-31 enrichment. Moreover, circ_0084043 knockdown impeded tumor growth *in vivo* and suppressed the level of Glut1 by modulating miR-31 and KLF3. Circ_0084043 promoted cell proliferation and glycolysis, and blocked apoptosis through the circ_0084043–miR-31–KLF3 regulatory axis in melanoma.

## 1 Introduction

Melanoma, originating from the malignant transformation of melanocytes, is the most massive malignant tumor in a number of skin cancer–related deaths [1,2]. As an extremely aggressive skin cancer, the mortality rate of melanoma is invariably high. Unfortunately, the incidence with melanoma has increased in recent years [3,4]. Despite significant advances in the diagnosis and treatment with molecular-targeted therapies and immunotherapy in recent years [5,6], the prognosis of melanoma patients is less than ideal and the 5-year survival rate is tragic [3,7]. Therefore, there is an urgent need to discover the molecular mechanisms of melanoma tumorigenesis and development and to identify biomarkers for the diagnosis and treatment of melanoma.

Recently, the crucial role of circular RNAs (circRNAs) in numerous human diseases, including cancers, has attracted wide attention [8]. CircRNAs are a class of noncoding RNAs that have more stable and conservative properties than linear RNAs because there are no 3′-poly (A) tail and 5′-ends [9,10]. CircRNAs have many biological functions, including adjustment of alternative splicing, regulation of parental gene expression, combination with RNA-binding proteins, and their role as microRNA (miRNA) sponges [11]. Several circRNAs have been documented to be associated with melanoma. For example, Yang et al. identified a series of dysregulated circRNAs through the microarray analysis of uveal melanoma tissues and normal tissues, including hsa_circ_0119873, hsa_circ_0128533, and hsa_circ_0047924 [12]. Also, Wang et al. obtained several differently expressed circRNAs from melanoma cells and normal melanocytes by microarray analysis [13]. These data indicated that circRNAs are implicated in the development of melanoma. A previous study identified that circ_0084043 was significantly upregulated in melanoma [14], which attracted our attention. However, the exact role of circ_0084043 in melanoma was not fully elucidated, and more associated mechanisms of action of circ_0084043 need to be explored.

CircRNAs are located mainly in the cytoplasm, which lays a foundation for circRNAs to act as competing endogenous RNAs of miRNAs [15,16]. MiRNAs are a kind of noncoding RNAs with 18–22 nucleotides in length. The crucial role of miR-31 is documented in various types of cancers, such as breast cancer, triple negative breast cancer, and colon cancer [17,18,19]. Unfortunately, the role of miR-31 in melanoma was rarely reported, and the associated mechanism of action is worthy of investigation. Generally, miRNAs function by combining with the 3′-UTR of targeted mRNAs to suppress their expression [20]. Krüppel-like factor 3 (KLF3), which belongs to the Krüppel-like factor family of zinc finger transcription factors, is located at chromosome 4q14 [21]. Research has implicated KLF3 in the regulation of adipogenesis, hematopoiesis, and muscle cell biology [22,23,24]. Recently, the involvement of KLF3 in the progression of cancers, particularly lung cancer, has been partly identified [25]. KLF3 was usually identified as a target of miRNAs. However, the potential role of KLF3 in cancers is not clear and needs to be explored.

Here, the abundance of circ_0084043 was determined in melanoma tissues and cells, and the function of circ_0084043 was investigated both *in vitro* and *in vivo*. Besides, the underlying mechanism of action was explored by analyzing the downstream miRNA and mRNA. The objective of our research was to determine the role of circ_0084043 in melanoma and provide a novel mechanism of action so as to further understand the progression of melanoma.

## 2 Materials and methods

### 2.1 Specimen collection

A total of 32 melanoma tissues and matched non-tumor tissues were obtained from the First Affiliated Hospital of University of South China. All tissues were frozen in liquid nitrogen and preserved at −80°C.


**Informed consent:** Informed consent has been obtained from all individuals included in this study.
**Ethical approval:** The research related to human use has been complied with all the relevant national regulations, institutional policies and in accordance with the tenets of the Helsinki Declaration, and has been approved by the Ethics Committee of the First Affiliated Hospital of University of South China.

### 2.2 Cell lines and cell culture

Melanoma cell lines (A375 and A875) were purchased from BeNa Culture Collection (Suzhou, China). Human epidermal melanocytes (HEMn-LP) were obtained from Gibco (Carlsbad, CA, USA). All cells were maintained in DMEM culture medium (Gibco) with 10% fetal bovine serum (Gibco) at 37°C under 5% CO_2_.

### 2.3 Cell transfection

The oligonucleotides and plasmids, including small interfering RNA (siRNA) targeting circ_0084043 (si-circ_0084043; 5′-UAAGCUUACAGGUACUUCCUU-3′), short hairpin RNA (shRNA) targeting circ_0084043 (sh-circ_0084043; 5′-TTTCATTGTATGTAGGTCC-3′), overexpression vector pcDNA-KLF3, and corresponding negative controls (si-NC, sh-NC, and pcDNA-Control), were assembled by GenePharma (Shanghai, China). MiR-31 mimic, miR-31 inhibitor, and relative negative controls (miRNA NC and inhibitor NC) were customized by RiboBio (Guangzhou, China). These oligonucleotides or plasmids were inserted into cells using Lipofectamine 3000 (Invitrogen, Carlsbad, CA, USA). The transfected cells were cultivated for 48 h and used for subsequent tests.

### 2.4 qRT-PCR analysis

Trizol reagent (Invitrogen) was utilized to obtain total RNA from melanoma tissues and cells. Then a HiScript III First Strand cDNA Synthesis Kit (Vazyme, Nanjing, China) or a miRNA First Strand Synthesis Kit (Vazyme) was used to conduct the reverse transcription reaction. Next, the amplification reaction was performed using SYBR Mix (Vazyme) and a CFX96 Touch Deep Well Real-Time PCR Detection System (Bio-Rad, Hercules, CA, USA). The relative expression was analyzed using the 2^−ΔΔCt^ method and standardized by GAPDH or U6. The primer sequences were as follows: circ_0084043, F: 5′-TTCTAGACAGCCGGGGAGTG-3′ and R: 5′-CCAAAACCTTTCTTTCTTGATGGGA-3′; miR-31, F: 5′-GCCGCAGGCAAGATGCTGGC-3′ and R: 5′-CAGTGCAGGGTCCGAGGT-3′; KLF3, F: 5′-TGTCTCAGTGTCATACCCATCT-3′ and R: 5′-CCTTCTGGGGTCTGAAAGAACTT-3′; GAPDH, F: 5′-ACCACAGTCCATGCCATCAC-3′ and R: 5′-TCCACCACCCTGTTGCTGTA-3′; U6, F: 5′-GCGCGTCGTGAAGCGTTC-3′ and R: 5′-GTGCAGGGTCCGAGGT-3′.

### 2.5 MTT assay

A375 and A875 cells with different transfections were placed into 96-well plates (5 × 10^3^ cells/well). Then 10 µL of MTT solution (Beyotime, Shanghai, China) was pipetted into each well at different time points (0, 24, 48, and 72 h) to incubate cells for another 4 h at 37°C. Afterward, dimethyl sulfoxide (DMSO; Beyotime) was pipetted into each well to dissolve the formazan. The absorbance at 490 nm was ascertained using an iMark microplate reader (Bio-Rad).

### 2.6 Flow cytometry assay

A375 and A875 cells with different transfections in 6-well plates were trypsinized and washed using PBS. Then, the cells (5 × 10^4^) were stained with an Annexin V-FITC/PI Apoptosis Detection Kit (Vazyme) following the manufacturer’s instructions. Cells at different time points were analyzed using a flow cytometer (BD Biosciences, San Jose, CA, USA).

### 2.7 Detection of glucose uptake, lactate production, and ATP level

Glucose uptake, lactate production, and ATP level were detected to determine the progression of glycolysis. Glucose consumption, lactate production, and ATP level were evaluated using a Glucose Assay Kit (BioVision, Milpitas, CA, USA), Lactate Assay Kit (BioVision), and ATP Assay Kit (Beyotime), respectively.

### 2.8 Target prediction and verification

Bioinformatics tools, including TargetScan (http://www.targetscan.org/vert_72/) and CircInteractome (https://circinteractome.nia.nih.gov/miRNA_Target_Sites/mirna_target_sites.html), were applied to predict the potential targets.

The relationship between miR-31 and circ_0084043 or KLF3 was further validated by the dual-luciferase reporter assay. To be specific, partial wild-type (WT, harboring the specific binding site for miR-31) and mutant-type (MUT, harboring the mutational binding site for miR-31) circ_0084043 sequences were amplified and inserted into the pGL4 vector (Promega, Madison, WI, USA), namely, WT-circ_0084043 and MUT-circ_0084043. Likewise, the 3′-UTR of KLF3 (WT and MUT) were also cloned into the pGL4 vector to generate WT-KLF3 3′-UTR and MUT-KLF3 3′-UTR. These fusion plasmids were transfected into A375 and A875 cells along with miR-31 mimic or miRNA NC. After 48 h, the firefly luminescence intensity was measured using the Dual-Luciferase Reporter Assay System (Promega) and normalized by Renilla luminescence activity.

### 2.9 Western blot

Western blot was carried out as previously described elsewhere [26]. Briefly, the proteins were separated and transferred to PVDF membranes. Subsequently, the membranes were blocked with 5% skim milk and probed with the primary antibodies against KLF3 (1:1,000; ab154531; Abcam, Cambridge, MA, USA), glucose transporter 1 (Glut1) (1:100,000; ab115730; Abcam) and GAPDH (1:2500; ab9485; Abcam) and with the corresponding secondary antibody (1:5,000; ab205718; Abcam).

### 2.10 Tumor formation assay *in vivo*

Experimental mice (BALB/c, 6-week-old), from Shanghai SIPPR-BK Laboratory Animal Co. Ltd (Shanghai, China), were divided into two groups (*n* = 6). A375 cells with sh-circ_0084043 or sh-NC transfection were subcutaneously inoculated into the left flank of mice back. From the seventh day after inoculation, the tumor volume (length × width^2^ × 0.5) in the sh-circ_0084043 group and in the sh-NC group was measured every 4 days. All mice were sacrificed after 27 days. Tumor tissues were excised for weighing and expression analysis.


**Ethical approval:** The research related to animal use has been complied with all the relevant national regulations and institutional policies for the care and use of animals and has been approved by the Animal Ethics Committee of the First Affiliated Hospital of University of South China.

### 2.11 Data processing

All experiments consisted of at least three independent repetitions. Statistical analysis was performed using SPSS software (SPSS Inc., Chicago, IL, USA), and the data were expressed as mean ± standard deviation. One-way analysis of variance or Student’s *t*-test was employed to conduct differential analysis. The difference was considered statistically significant at *P* < 0.05.

## 3 Results

### 3.1 Circ_0084043 was aberrantly overexpressed in melanoma tissues and cells

**Figure 1:**
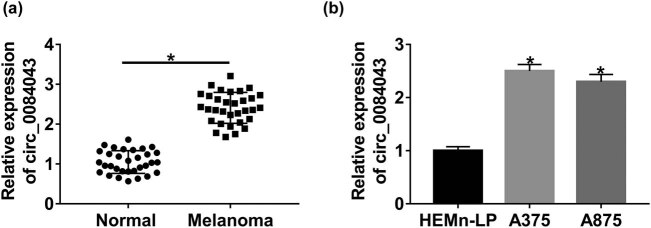
Circ_0084043 was highly regulated in melanoma tissues and cell lines. (a) The abundance of circ_0084043 in melanoma tissues (*n* = 32) and normal nontumor tissues (*n* = 32) was detected by qRT-PCR. (b) The abundance of circ_0084043 in melanoma cell lines (A375 and A875) and HEMn-LP cells was detected by qRT-PCR. *P < 0.05.

qRT-PCR analysis indicated the abundance of circ_0084043 in cancer tissues and cells. Noticeably, the expression of circ_0084043 was higher in melanoma tissues (*n* = 32) than in normal nontumor tissues (*n* = 32) (Figure 1a). Also, the expression of circ_0084043 was significantly elevated in melanoma cell lines (A375 and A875) compared with that in HEMn-LP cells (Figure 1b). The data suggested that the dysregulation of circ_0084043 might be associated with the development of melanoma.

### 3.2 Circ_0084043 knockdown impaired cell proliferation, induced cell apoptosis, and inhibited glycolysis in melanoma cells

**Figure 2:**
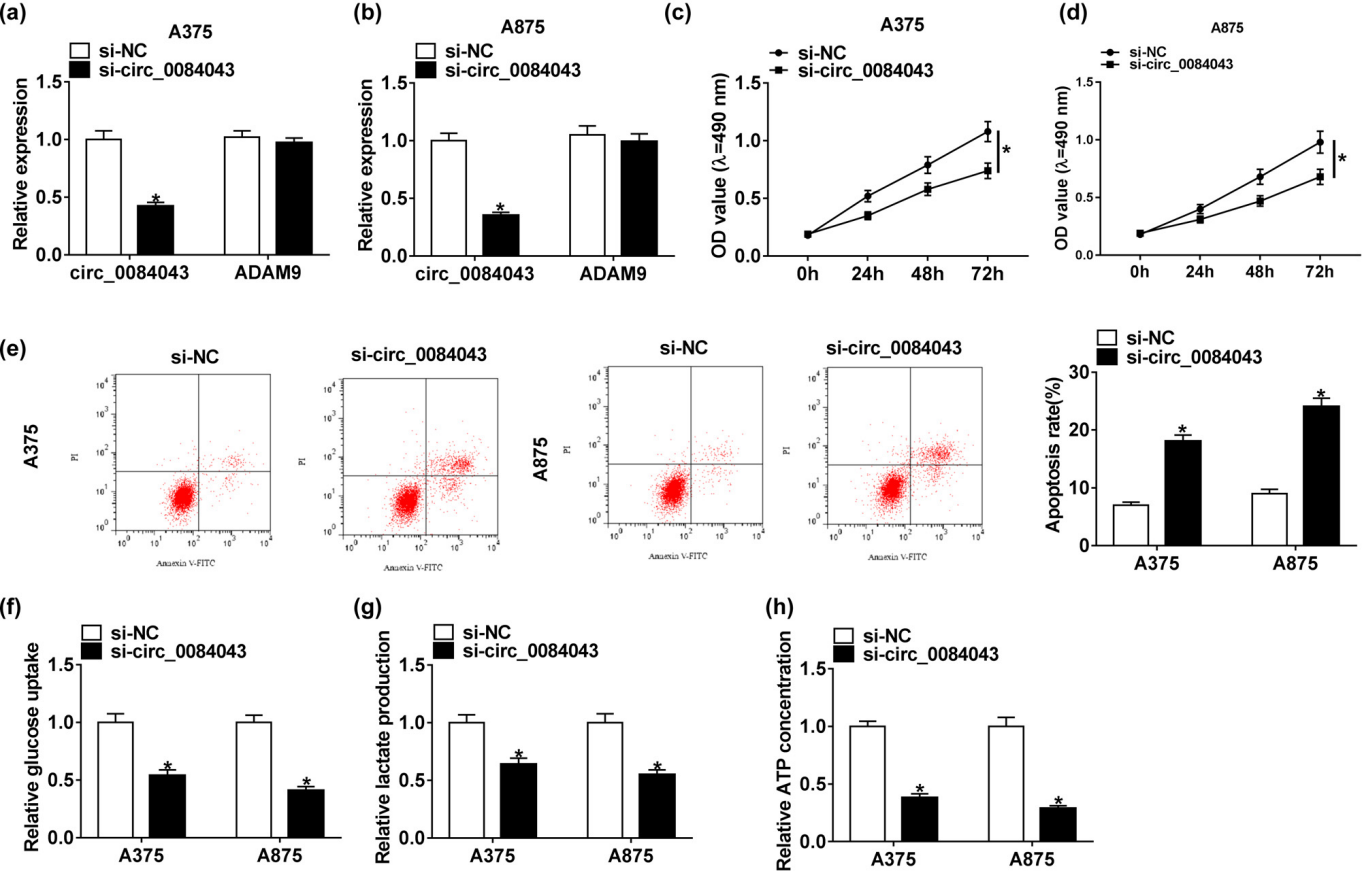
Circ_0084043 knockdown inhibited proliferation and glycolysis but induced apoptosis in melanoma cells. A375 and A875 cells were transfected with si-circ_0084043 or si-NC. (a and b) The expression of circ_0084043 was measured by qRT-PCR. (c and d) Cell proliferation was assessed by the MTT assay. (e) Cell apoptosis was examined by the flow cytometry assay. (f–h) The progression of glycolysis was evaluated, according to glucose consumption, lactate production, and ATP concentration. *P < 0.05.

The endogenous content of circ_0084043 was knocked down in A375 and A875 cells by transfecting with si-circ_0084043 to determine the role of circ_0084043. First, we noticed that the expression of circ_0084043 was clearly decreased in cells with si-circ_0084043 transfection, while the expression of its pre-mRNA (ADAM9) differed little in cells with si-circ_0084043 transfection relative to si-NC (Figure 2a and b). Then, the MTT assay indicated that cell proliferation was notably reduced in cells transfected with si-circ_0084043 relative to si-NC (Figure 2c and d). Next, flow cytometry assay manifested that circ_0084043 knockdown increased the rate of apoptosis (Figure 2e). Moreover, the levels of glucose consumption, lactate production, and ATP concentration were markedly declined in A375 and A875 cells transfected with si-circ_0084043 compared with si-NC (Figure 2f–h). These analyses suggested that circ_0084043 knockdown impaired proliferation and glycolysis but promoted apoptosis in melanoma cells.

### 3.3 MiR-31 was a target of circ_0084043, and miR-31 inhibition reversed the impact of circ_0084043 knockdown in melanoma cells

**Figure 3:**
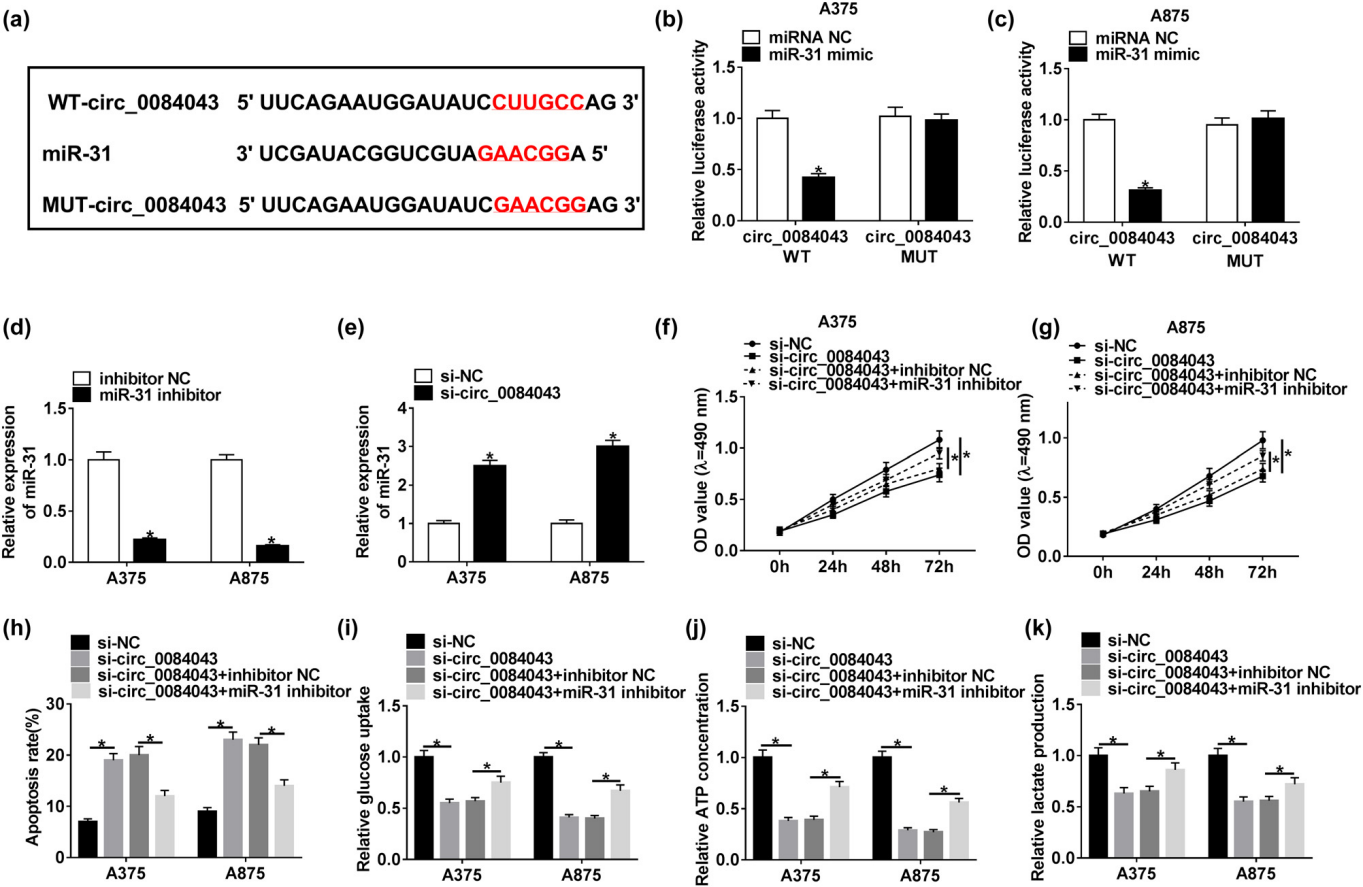
MiR-31 was targeted by circ_0084043, and its inhibition reversed the effects of circ_0084043 knockdown. (a) The binding site between miR-31 and circ_0084043 was analyzed by the online tool CircInteractome. (b and c) The relationship between miR-31 and circ_0084043 was verified by the dual-luciferase reporter assay. (d and e) The expression of miR-31 in the cells transfected with si-circ_0084043 or miR-31 inhibitor. (f and g) Cell proliferation, (h) cell apoptosis, and (i–k) the progression of glycolysis were detected by the MTT assay, flow cytometry assay, and the levels of glucose consumption, lactate production, and ATP concentration, respectively. *P < 0.05.

To detect whether circ_0084043 functioned by targeting its downstream miRNAs, the putative target miRNAs of circ_0084043 were predicted by the online database CircInteractome (Table A1). The partial sequences of circ_0084043 harboring the binding site for miR-31 were amplified and cloned into the pGL4 vector, named WT-circ_0084043. Meanwhile, the mutated sequences were also cloned into the pGL4 vector, named MUT-circ_0084043 (Figure 3a). As shown in Figure 3b and c, the miR-31 mimic prominently diminished the luciferase activity in A375 and A875 cells transfected with WT-circ_0084043 rather than with MUT-circ_0084043. In addition, the expression of miR-31 was obviously enhanced by circ_0084043 knockdown (Figure 3e). Next, the miR-31 expression was inhibited in A375 and A875 cells to observe its role. The transfection efficiency showed that the expression of miR-31 pronouncedly declined in A375 and A875 cells with miR-31 inhibitor transfection (Figure 3d). Furthermore, the cell proliferation inhibited by circ_0084043 knockdown was recovered by miR-31 inhibition (Figure 3f and 3g). However, the apoptosis rate increased in cells with si-circ_0084043 transfection but decreased in cells with si-circ_0084043 + miR-31 inhibitor transfection (Figure 3h). Unsurprisingly, si-circ_0084043 + miR-31 inhibitor transfection restored the glucose consumption, lactate production, and ATP concentration inhibited by si-circ_0084043 transfection (Figure 3i–k). These data indicated that circ_0084043 knockdown inhibited melanoma progression by modulating miR-31 *in vitro*.

### 3.4 KLF3 was targeted by miR-31, and KLF3 upregulation overturned the role of miR-31 mimic in melanoma cells

**Figure 4:**
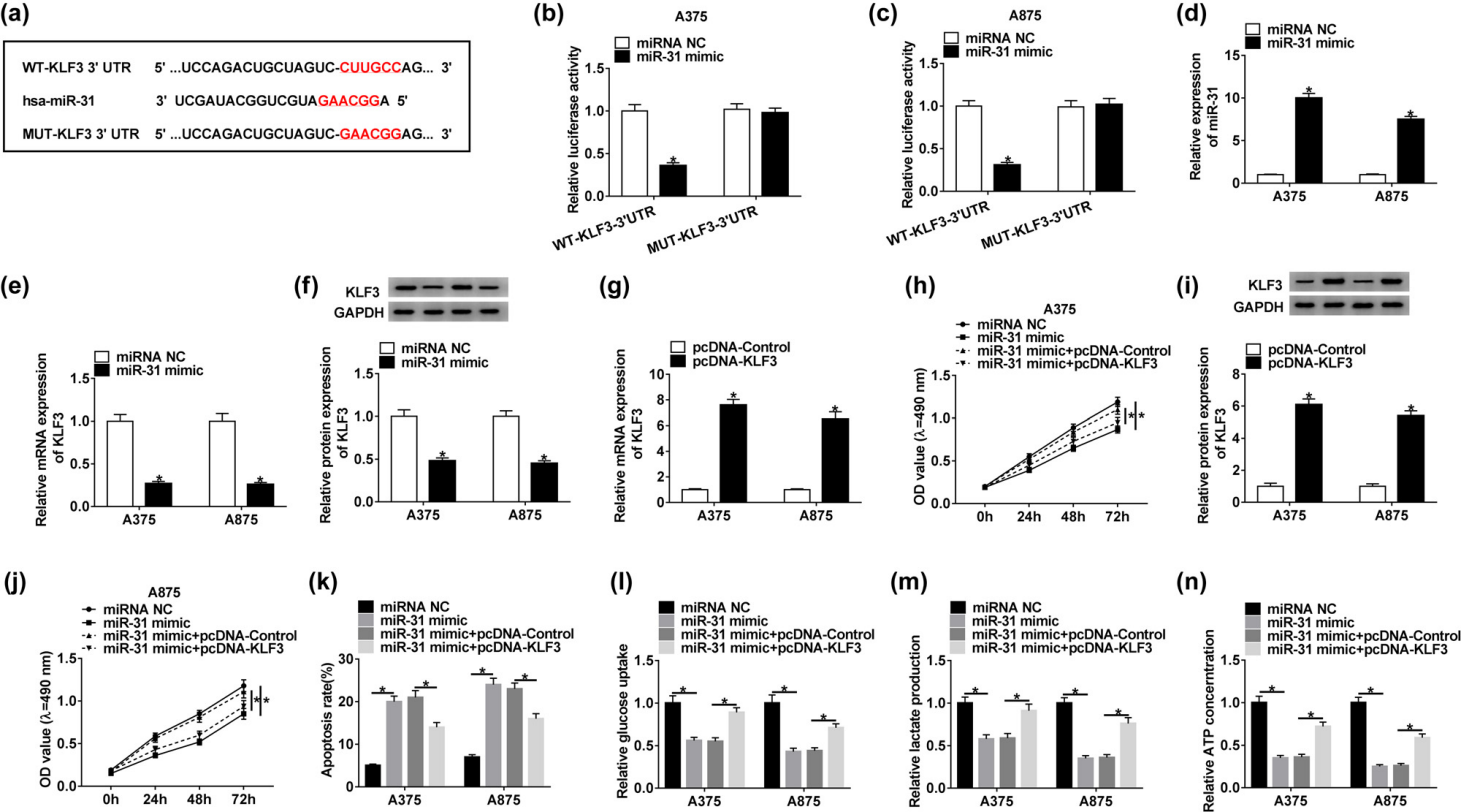
KLF3 was targeted by miR-31, and its overexpression abolished the role of miR-31 mimic. (a) The binding site between miR-31 and KLF3 3′-UTR was forecasted by the online tool TargetScan. (b and c) The interaction between miR-31 and KLF3 was confirmed by the dual-luciferase reporter assay. (d–f) The expression of miR-31 and KLF3 was checked in the cells transfected with miR-31 mimic. (g and i) The expression of KLF3 at mRNA and protein levels in the cells transfected with pcDNA-KLF3 was examined by qRT-PCR and western blot. (h and j) Cell proliferation was assessed by the MTT assay. (k) Cell apoptosis was examined by the flow cytometry assay. (l–n) The progression of glycolysis was evaluated, according to glucose consumption, lactate production, and ATP concentration. *P < 0.05.

Generally, miRNAs function by uniting with the 3′-UTR of targeted mRNAs. To determine whether miR-31 exerted its role by targeting downstream mRNAs, the potential mRNAs targeted by miR-31 were screened by the online tool TargetScan (Table A2). Subsequently, the sequences of KLF3 3′-UTR containing the binding site for miR-31 and the corresponding mutant sequences were cloned into the pGL4 vector to generate WT-KLF3 3′-UTR and MUT-KLF3 3′-UTR fusion plasmids (Figure 4a). We found that miR-31 mimic significantly decreased the luciferase activity in A375 and A875 cells introduced with WT-KLF3 3′-UTR but not with MUT-KLF3 3′-UTR (Figure 4b and c). In A375 and A875 cells transfected with miR-31 mimic, the expression of miR-31 was clearly increased (Figure 4d), indicating that the transfection efficiency was competent. Then, we also detected that the KLF3 expression in A375 and A875 cells transfected with miR-31 mimic was obviously impaired at both mRNA and protein levels (Figure 4e and f), suggesting that miR-31 overexpression suppressed the KLF3 expression. The detection of transfection efficiency showed that the expression of KLF3 was substantially elevated in A375 and A875 cells transfected with pcDNA-KLF3 (Figure 4g and i), suggesting that these transfected cells were available. Functionally, the MTT assay showed that the cell proliferation was inhibited in cells introduced with miR-31 mimic but reinforced in cells introduced with miR-31 mimic + pcDNA-KLF3 (Figure 4h and j). Inversely, the apoptosis rate was notably stimulated in cells transfected with the miR-31 mimic but repressed in cells transfected with miR-31 mimic + pcDNA-KLF3 (Figure 4k). Moreover, glucose consumption, lactate production, and ATP concentration were reduced by miR-31 enrichment but elevated by KLF3 overexpression (Figure 4l–n). These data indicated that miR-31 participated in proliferation, apoptosis, and glycolysis by regulating the expression of KLF3 in melanoma cells.

### 3.5 KLF3 overexpression rescued the effects of circ_0084043 knockdown in melanoma cells

**Figure 5:**
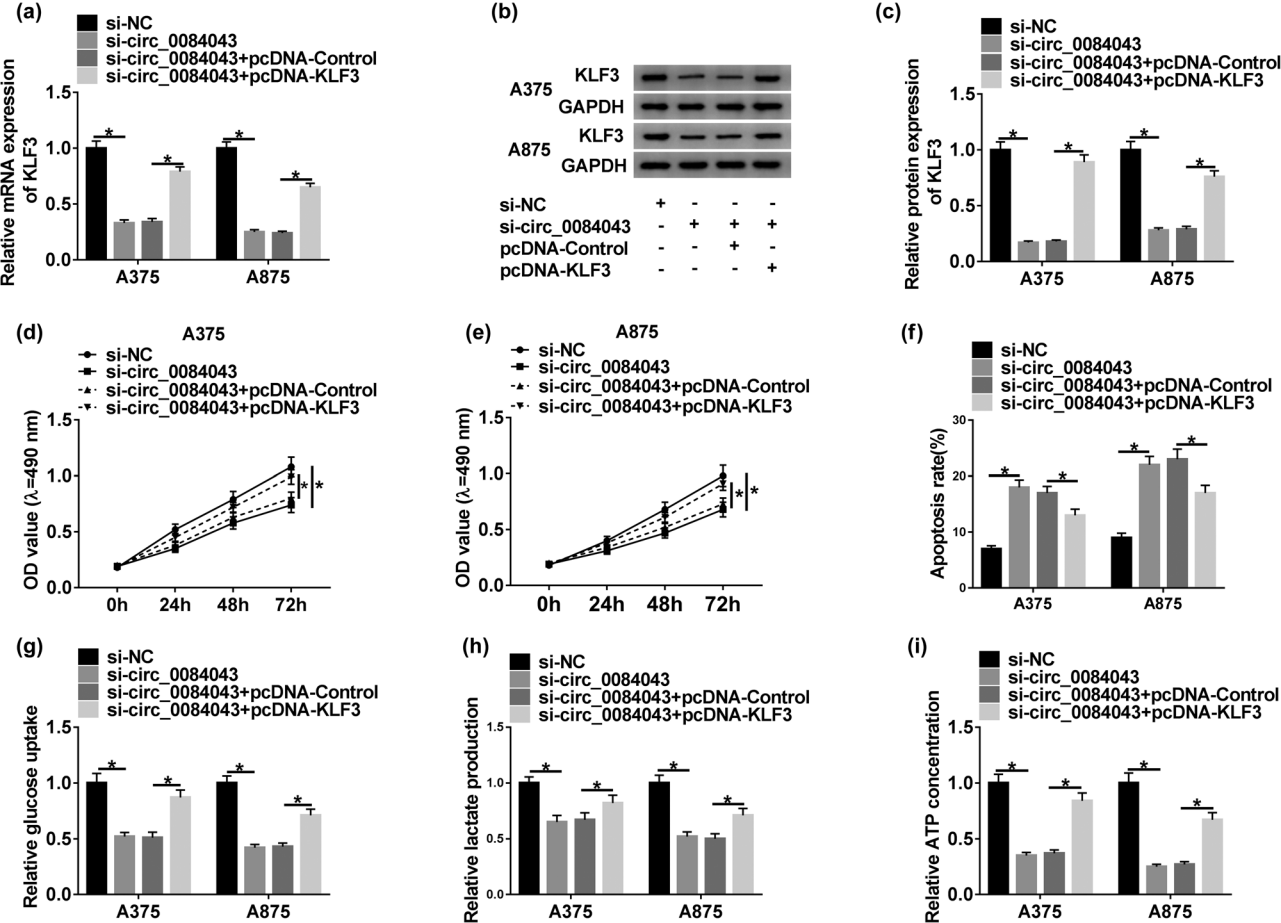
KLF3 overexpression rescued the role of circ_0084043 knockdown. A375 and A875 cells were transfected with si-circ_0084043 or si-circ_0084043 + pcDNA-KLF3, si-NC or si-circ_0084043 + pcDNA-Control as the control. (a–c) The expression of KLF3 at mRNA and protein levels was determined by qRT-PCR and western blot. (d and e) Cell proliferation was assessed by the MTT assay. (f) Cell apoptosis was examined by the flow cytometry assay. (g–i) The progression of glycolysis was evaluated, according to glucose consumption, lactate production, and ATP concentration. *P < 0.05.

To explore the interaction between circ_0084043 and KLF3, rescue experiments were performed. A375 and A875 cells were introduced with si-circ_0084043 or si-circ_0084043 + pcDNA-KLF3, with si-NC or si-circ_0084043 + pcDNA-Control as the control. The transfection efficiency was examined. We found that the expression of KLF3 at both mRNA and protein levels was decreased in cells with si-circ_0084043 transfection but regained in cells with si-circ_0084043 + pcDNA-KLF3 transfection (Figure 5a–c). Cell proliferation inhibited by si-circ_0084043 was elevated by si-circ_0084043 + pcDNA-KLF3 (Figure 5d and e). The apoptosis rate was induced by si-circ_0084043 transfection but suppressed by si-circ_0084043 + pcDNA-KLF3 transfection (Figure 5f). Additionally, KLF3 overexpression restored the glucose consumption, lactate production, and ATP concentration blocked by circ_0084043 knockdown (Figure 5g–i). The data indicated that circ_0084043 regulated cell proliferation, apoptosis, and glycolysis by promoting the KLF3 expression in melanoma cells.

### 3.6 Circ_0084043 regulated the expression of KLF3 by sponging miR-31

**Figure 6:**
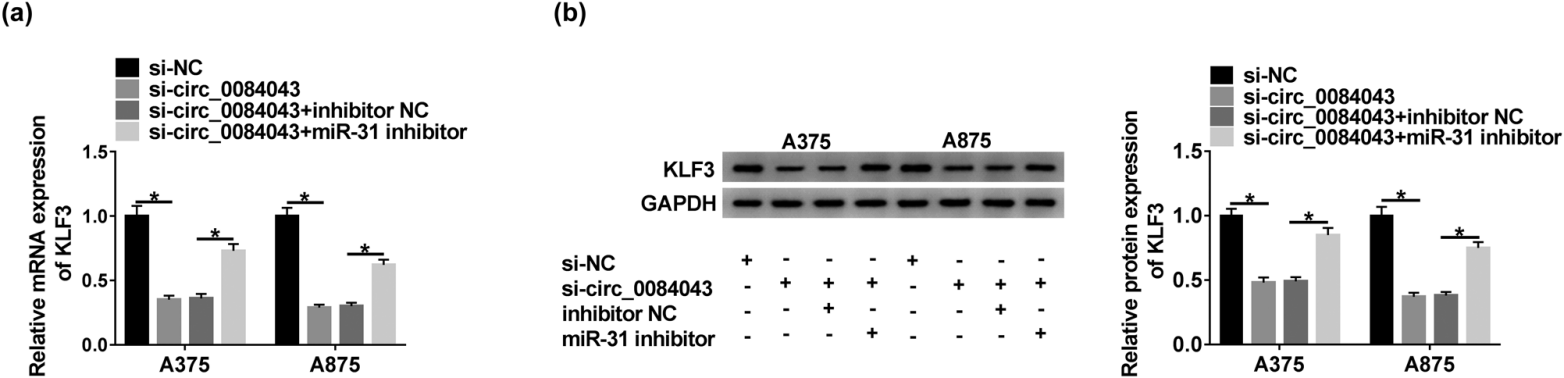
Circ_0084043 regulated the expression of KLF3 by targeting miR-31. A375 and A875 cells were transfected with si-circ_0084043 or si-circ_0084043 + miR-31 inhibitor, si-NC or si-circ_0084043 + inhibitor NC as the control. (a and b) The expression of KLF3 at mRNA and protein levels was quantified by qRT-PCR and western blot. *P < 0.05.

A375 and A875 cells were transfected with si-circ_0084043, si-NC, si-circ_0084043 + miR-31 inhibitor, and si-circ_0084043 + inhibitor NC. Apparently, the expression of KLF3 was inhibited in cells with si-circ_0084043 transfection relative to si-NC, while the expression of KLF3 was recovered in cells with si-circ_0084043 + miR-31 inhibitor transfection relative to si-circ_0084043 + inhibitor NC at both mRNA and protein levels (Figure 6a and b). The data suggested that KLF3 was regulated by circ_0084043 through miR-31.

### 3.7 Circ_0084043 knockdown inhibited tumorigenesis *in vivo*

**Figure 7:**
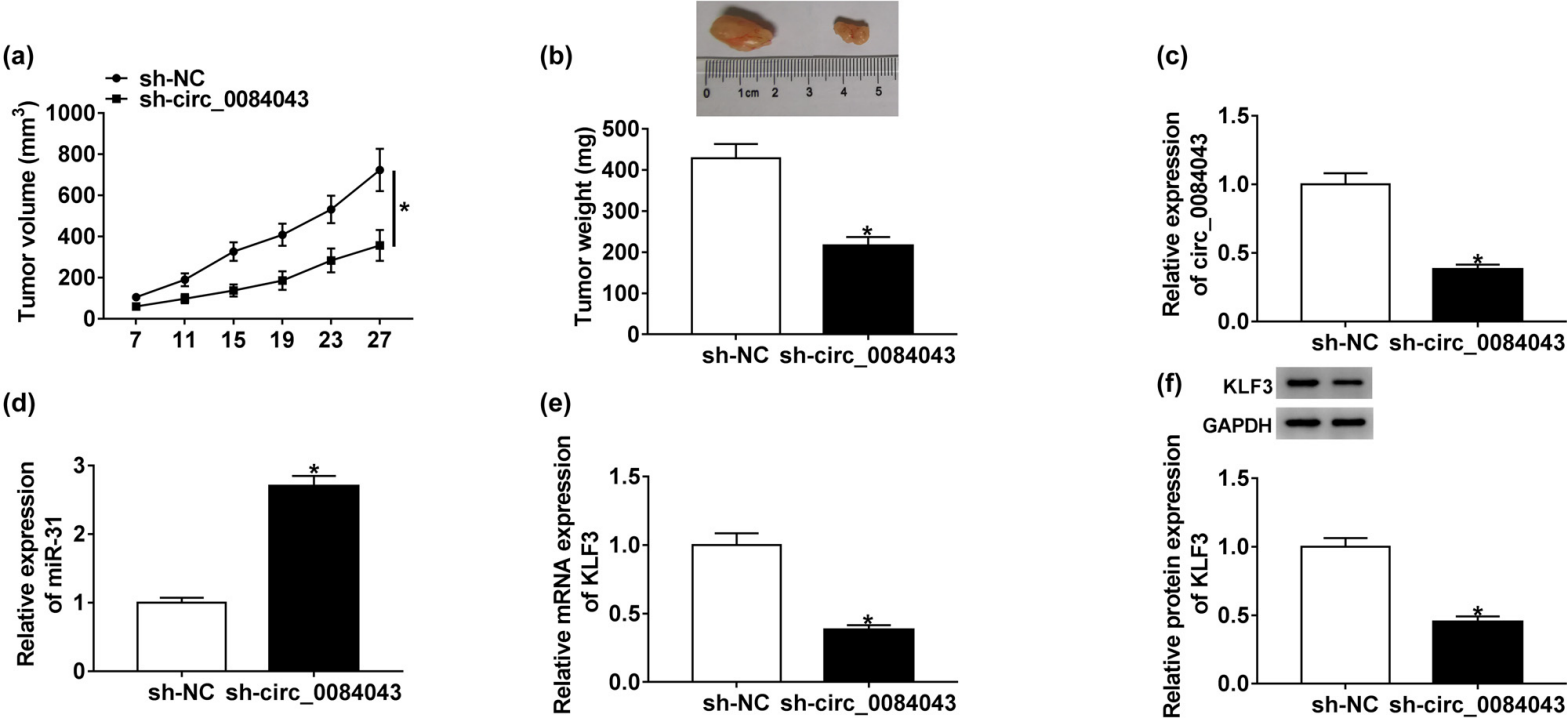
Circ_0084043 knockdown inhibited tumor growth *in vivo*. (a and b) Circ_0084043 knockdown significantly reduced the tumor volume and tumor weight. (c and d) The expression of circ_0084043 and miR-31 in removed tumor tissues was detected by qRT-PCR. (e and f) The expression of KLF3 in removed tumor tissues at both mRNA and protein levels was determined by qRT-PCR and western blot. *P < 0.05.

To ascertain the role of circ_0084043 *in vivo*, the xenograft model was established. A375 cells with sh-circ_0084043 transfection were inoculated in the right flank of mice groin, with sh-NC as the control. The tumor volume and tumor weight in the sh-circ_0084043 group were obviously lower than those in the sh-NC group (Figure 7a and b). Subsequently, the expression levels of circ_0084043, miR-31, and KLF3 in the removed tumor tissues were examined. The results displayed that the expression of circ_0084043 was lower in the sh-circ_0084043 group than in the sh-NC group, while the expression of miR-31 was enhanced in the sh-circ_0084043 group (Figure 7c and d). Moreover, the expression of KLF3 was declined in the sh-circ_0084043 group relative to that in the sh-NC group (Figure 7e and f). The data indicated that circ_0084043 knockdown impeded tumor growth *in vivo*.

### 3.8 Circ_0084043 modulated the expression of Glut1 through the miR-31/KLF3 axis

**Figure 8:**
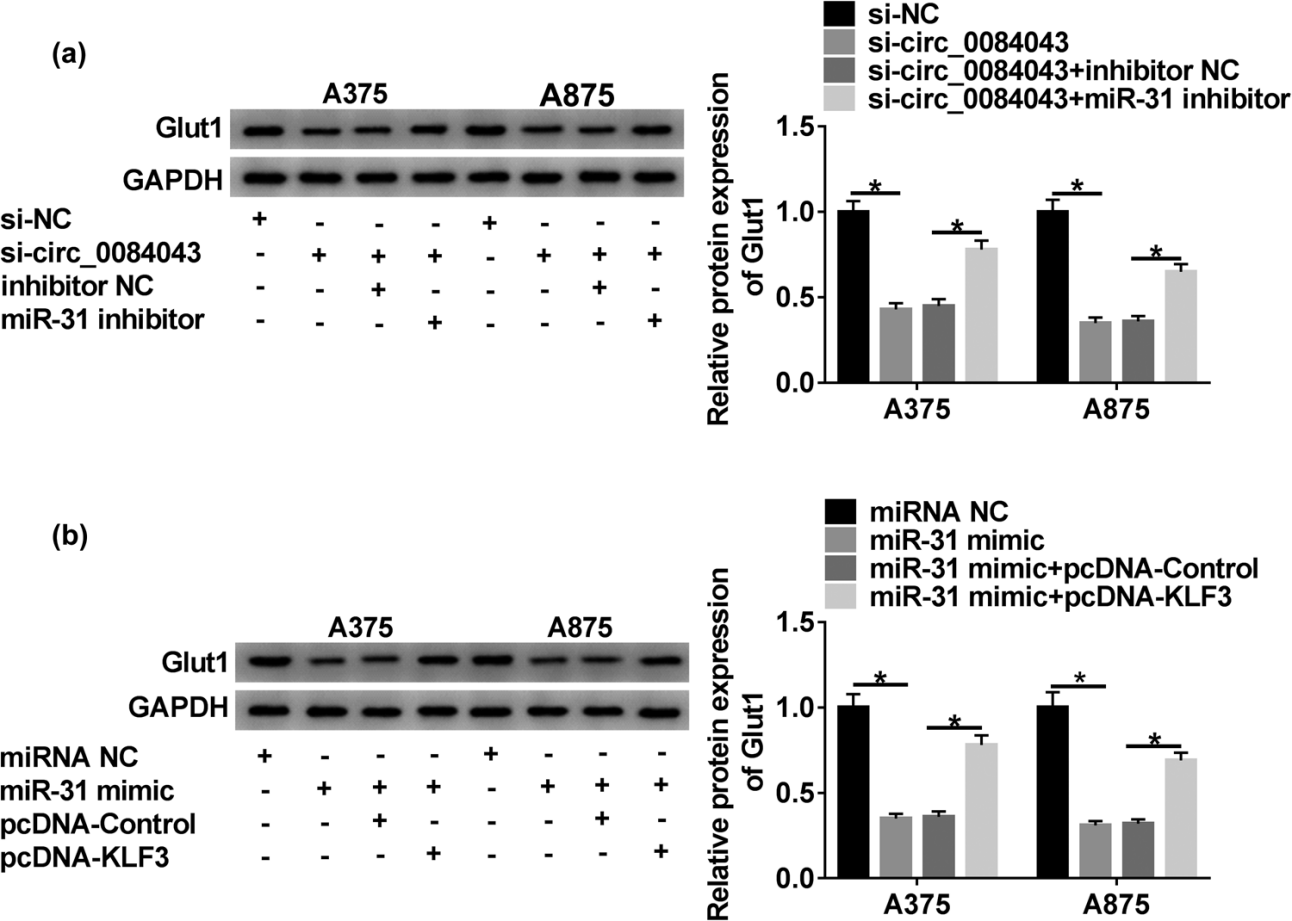
The expression of Glut1 was modulated by the circ_0084043/miR-31/KLF3 regulatory axis. (a) The protein level of Glut1 in A375 and A875 cells transfected with si-circ_0084043, si-NC, si-circ_0084043 + miR-31 inhibitor, or si-circ_0084043 + inhibitor NC was measured by western blot. (b) The protein level of Glut1 in A375 and A875 cells transfected with miR-31 mimic, miRNA NC, miR-31 mimic + pcDNA-KLF3, or miR-31 mimic + pcDNA-Control was measured by western blot. *P < 0.05.

Glut1 is a key regulator of glycolysis and is closely related to the occurrence and progression of malignant tumors. The expression of Glut1 was reduced in A375 and A378 cells with si-circ_0084043 transfection but recovered in cells with si-circ_0084043 + miR-31 inhibitor transfection at the protein level (Figure 8a). Besides, the expression of Glut1 was reduced by the miR-31 mimic but enhanced by KLF3 overexpression (Figure 8b). The data pointed out that circ_0084043 facilitated the expression of Glut1 via the modulation of the miR-31/KLF3 axis.

## 4 Discussion

Increasing evidence shows that melanoma is a severe threat to people’s health. It is essential to find novel therapeutic strategies against melanoma. In our study, we discovered that circ_0084043 was abundantly expressed in melanoma tissues and cells, and its downregulation inhibited proliferation and glycolysis but triggered apoptosis in melanoma cells. We also screened and identified the target miRNA (miR-31) of circ_0084043 and the target mRNA (KLF3) of miR-31, revealing a potential role of circ_0084043 in the progression of melanoma.

CircRNAs are involved in the progression of many types of cancers, whereas knowledge of their specific role is limited and inadequate. Using circRNA microarray analysis, a previous study claimed that circ_0084043 was aberrantly upregulated in melanoma tissues and that reintroduction of circ_0084043 stimulated cell proliferation and metastasis in melanoma [14]. Consistent with this research, we also discovered that circ_0084043 was highly expressed in melanoma tissues and cells. Functional analysis revealed that circ_0084043 knockdown restrained cell proliferation, induced cell apoptosis, and impeded glycolysis. This is the first study to explore the regulation of circ_0084043 in glycolysis, and we proposed that circ_0084043 promoted glycolysis metabolism to promote melanoma development. Moreover, circ_0084043 knockdown suppressing tumor growth was also confirmed *in vivo*. Accumulating evidence has shown that circRNAs participate in the progression of cancers through various mechanisms, such as interaction with miRNAs, translation into proteins, and generation of fusion circRNAs [10,11,27]. The above-mentioned study also found that circ_0084043 served as an oncogene in melanoma by functioning as a molecular sponge of miR-153-3p [14]. To determine the novel mechanisms of circ_0084043 function in melanoma, the downstream miRNAs of circ_0084043 were predicted and screened.

MiR-31 was a target of circ_0084043 with a specific binding site. Besides, the expression of miR-31 was strikingly reduced in melanoma cell lines. Given that the role of miR-31 in melanoma was partly mentioned in preceding studies, miR-31 was thus chosen as a research object in this study. Throughout the former studies, the endogenous expression of miR-31 was consistently reduced in melanoma tissues and cells [28,29,30]. Moreover, miR-31 overexpression blocked tumor growth and chemoresistance of melanoma [30]. In agreement with these studies, we observed that miR-31 enrichment attenuated cell proliferation and glycolysis but enhanced cell apoptosis, and miR-31 inhibition reversed the regulatory effects of circ_0084043 knockdown. These findings emphasized that miR-31 was a tumor suppressor in melanoma.

The circRNA–miRNA–mRNA axis is an indispensable regulatory way that modulates cancer-related biological processes [31]. In our study, KLF3 was verified to be targeted by miR-31 through bioinformatics prediction and dual-luciferase reporter detection. Interestingly, the role of KLF3 varied in different types of cancers. For instance, KLF3 downregulation inhibited tumorigenesis and the development of lung cancer [24]. Inversely, loss of KLF3 led to malignant phenotypes and poor prognosis of colorectal cancer [32]. This might be due to the different expression patterns of KLF3 in different cancers. A previous study elucidated that KLF3 was highly expressed in melanoma cells and that KLF3 knockdown inhibited cell viability, migration, and invasion [33]. Similarly, we also found that KLF3 was upregulated in melanoma cell lines and that KLF3 overexpression abolished the effect of miR-31 enrichment on the activities of melanoma cells, suggesting that KLF3 was indeed an oncogene in melanoma.

Taken together, circ_0084043 expression was abnormally enhanced in melanoma tissues and cells. Circ_0084043 contributed to cell proliferation and glycolysis but inhibited cell apoptosis through the regulation of the circ_0084043–miR-31–KLF3 axis. Our study adds to the understanding of the progression of melanoma and provides a promising biomarker for the treatment of melanoma. The limitation of this study was that we mainly investigated the function of circ_0084043 by its knockdown, and the effects of circ_0084043 overexpression should be monitored in the future work.

## Appendix

**Table A1:** The partial target miRNAs of circ_0084043 predicted by CircInteractome

MiRNA name	Targeting sites	Score
hsa_circ_0084043 (5′…3′)	GAAGUGUGCCACUGGGAAUGCUU	77
hsa-miR-1179 (3′ …… 5′)	|||||||	
	GGUUGGUUACUUUCUUACGAA	
hsa_circ_0084043 (5′…3′)	AAUGGCAUUUGUGGGAACAGUGU	87
hsa-miR-1208 (3′…5′)	|||||||	
	AGGCGGACAGACUUGUCACU	
hsa_circ_0084043 (5′…3′)	UCUCCAGUGACACCUCCCAGAGA	99
hsa-miR-326 (3′…5′)	|||||||	
	GACCUCCUUCCCGGGUCUCC	
hsa_circ_0084043 (5′…3′)	UUCAGAAUGGAUAUCCUUGCCAG	82
hsa-miR-31 (3′…5′)	||||||	
	UCGAUACGGUCGUAGAACGGA	
hsa_circ_0084043 (5′…3′)	GGAGUGUUCCUCGACAUGUUUCU	89
hsa-miR-494 (3′…5′)	|||| |||||||	
	CUCCAAAGGGCACAUACAAAGU	
hsa_circ_0084043 (5′…3′)	UCUCUGGCAAUGAAUACAAGAAG	73
hsa-miR-578 (3′…5′)	|||||||	
	UGUUAGGAUCUCGUGUUCUUC	

**Table A2:** The partial target mRNAs of miR-31 predicted by TargetScan

Target gene	Representative transcript	Gene name
KLF3	ENST00000261438.5	Krüppel-like factor 3 (basic)
CEP85	ENST00000252992.4	Centrosomal protein 85 kDa
UBN1	ENST00000262376.6	Ubinuclein 1
AMOTL1	ENST00000317837.9	Angiomotin like 1
EIF5	ENST00000216554.3	Eukaryotic translation initiation factor 5
SLC30A6	ENST00000282587.5	Solute carrier family 30 (zinc transporter), member 6
RAB31	ENST00000578921.1	RAB31, member RAS oncogene family
NDRG3	ENST00000373803.2	NDRG family member 3
